# Targeting activating mutations of EZH2 leads to potent cell growth inhibition in human melanoma by derepression of tumor suppressor genes

**DOI:** 10.18632/oncotarget.4809

**Published:** 2015-08-12

**Authors:** Jessamy C. Tiffen, Dilini Gunatilake, Stuart J. Gallagher, Kavitha Gowrishankar, Anja Heinemann, Carleen Cullinane, Ken Dutton-Regester, Gulietta M. Pupo, Dario Strbenac, Jean Y. Yang, Jason Madore, Graham J. Mann, Nicholas K. Hayward, Grant A. McArthur, Fabian V. Filipp, Peter Hersey

**Affiliations:** ^1^ Melanoma Immunology and Oncology Group, Centenary Institute, University of Sydney, NSW, Australia; ^2^ Melanoma Research Group, Kolling Institute of Medical Research, University of Sydney, NSW, Australia; ^3^ Translational Research Laboratory, Peter MacCallum Cancer Centre, Melbourne, Victoria, Australia; ^4^ Oncogenomics Laboratory, QIMR Berghofer Medical Research Institute, Brisbane, Queensland, Australia; ^5^ Center for Cancer Research, University of Sydney at Westmead Millennium Institute, Westmead, NSW, Australia; ^6^ School of Mathematics and Statistics, University of Sydney, NSW, Australia; ^7^ Sydney Medical School, University of Sydney, NSW, Australia; ^8^ Melanoma Institute Australia, Crows Nest, Sydney, NSW, Australia; ^9^ Oncogenic Signalling and Growth Control Program, Peter MacCallum Cancer Centre, Melbourne, Victoria, Australia; ^10^ Systems Biology and Cancer Metabolism, Program for Quantitative Systems Biology, University of California Merced, Merced, USA

**Keywords:** melanoma, EZH2, H3K27me3, epigenetics, targeted therapy

## Abstract

The epigenetic modifier EZH2 is part of the polycomb repressive complex that suppresses gene expression via histone methylation. Activating mutations in EZH2 are found in a subset of melanoma that contributes to disease progression by inactivating tumor suppressor genes. In this study we have targeted EZH2 with a specific inhibitor (GSK126) or depleted EZH2 protein by stable shRNA knockdown. We show that inhibition of EZH2 has potent effects on the growth of both wild-type and EZH2 mutant human melanoma *in vitro* particularly in cell lines harboring the EZH2^Y646^ activating mutation. This was associated with cell cycle arrest, reduced proliferative capacity in both 2D and 3D culture systems, and induction of apoptosis. The latter was caspase independent and mediated by the release of apoptosis inducing factor (AIFM1) from mitochondria. Gene expression arrays showed that several well characterized tumor suppressor genes were reactivated by EZH2 inhibition. This included activating transcription factor 3 (ATF3) that was validated as an EZH2 target gene by ChIP-qPCR. These results emphasize a critical role for EZH2 in the proliferation and viability of melanoma and highlight the potential for targeted therapy against EZH2 in treatment of patients with melanoma.

## INTRODUCTION

Abnormal silencing of tumor suppressor genes by hypermethylation has long been recognized as a critical mediator of tumorigenesis [1]. This includes not only DNA methylation of CpG dinucleotides in gene promoter regions, but also methylation of histones which DNA is wound around to form chromatin [2]. These epigenetic modifications play a crucial role in the regulation of gene expression.

Enhancer of Zeste Homologue 2 (EZH2) is the catalytic subunit of the polycomb repressive complex 2 (PRC2), responsible for trimethylation of lysine 27 of H3 histones (H3K27me3) [3, 4]. This mark is predominantly associated with gene silencing via compaction of chromatin. However, a dual methylation and silencing mechanism exists in cancer, in which EZH2 is also able to recruit DNA methyltransferases (DNMTs) to direct DNA methylation [5, 6].

EZH2 is considered an oncogene due to its overexpression in many different types of cancer and its association with poor prognosis [7]. Additionally, somatic heterozygous mutations were first identified within the catalytic SET domain of EZH2 in lymphoma [8–10]. The amino acid substitution that occurs at a crucial tyrosine residue (Y641) within this domain changes the conformation of EZH2 and increases its H3K27 tri-methylation activity [11, 12]. It is proposed that in cancer, an abnormal accumulation of H3K27me3 represses the expression of tumor suppressors, including genes related to cell cycle inhibition, apoptosis, senescence and differentiation [13].

A growing body of evidence supports a role for EZH2 in the pathogenesis of melanoma. Somatic mutations in EZH2 occur in ~3% of melanoma, which to date is the only other cancer type that contains the same activating Y646 mutation as lymphoma (equivalent tyrosine based on current NCBI protein reference sequence; NP_004447.2) that is the most frequent alteration [14, 15]. In addition the importance of EZH2 dysregulation in melanoma is supported by its amplification in 5% of melanoma samples [14] and increased mRNA expression in 14% of cases in the TCGA dataset [14].

In Melanoma, knockdown of BRAF^V600E^ was shown to profoundly reduce the expression levels of EZH2 [16], suggesting that increased BRAF activity frequently found in melanoma may contribute to the abnormal overexpression of EZH2. Additionally, immunohistochemistry (IHC) studies have revealed an incremental increase in EZH2 protein levels from benign nevi to metastatic melanoma [17, 18]. High levels of EZH2 were associated with increased proliferation (Ki-67 staining), thicker primary melanomas, increased invasion and poor survival [15, 19].

Further evidence for the importance of EZH2 in melanoma came from knockdown studies of EZH2 that resulted in reduced proliferation, restoration of a senescent like phenotype and reduced growth of xenografts in mice, due to the reactivation of CDKN1A/p21 tumor suppressor [20]. Similarly, inducible knockdown of EZH2 in a transgenic melanoma mouse model was able to prevent melanoma growth and metastasis, in a manner comparable to the EZH2 inhibitor, GSK503 suggesting a role in melanoma progression [15]. Lastly, EZH2 overexpression has been implicated in dampening efficient T-cell mediated immune responses in uveal melanoma [21].

These studies point to EZH2 inhibition as a possible treatment in melanoma but have not focused on melanoma cells that harbor endogenous EZH2^Y646^ activating mutations, nor provided insights into the mechanism of EZH2 mediated cell growth inhibition in these mutants.

In the following study we used a small molecule inhibitor GSK126, to competitively inhibit EZH2's methytransferase activity, or depleted EZH2 protein with shRNA. GSK126 potently inhibited the growth of EZH2^Y646^ mutant cells and caused caspase independent apoptosis that was reliant on mitochondrial release of AIFM1 (apoptosis inducing factor, mitochondrion associated 1) protein. This was most likely caused by an imbalance of pro- and anti-apoptotic proteins that regulate mitochondrial membrane stability. Gene expression arrays demonstrated that treatment with GSK126 was associated with upregulation of diverse tumor suppressor genes which appear involved in the induction of apoptosis and inhibition of cell growth. This included ATF3, a transcription factor known to bind and activate the promotor of the gene encoding the pro-apoptotic protein Noxa [22]. Therefore blocking EZH2 activity may represent an effective strategy for preventing melanoma progression.

## RESULTS

### EZH2 and H3K27me3 are overexpressed in both EZH2 mutant and wild-type melanoma cell lines but EZH2^Y646^ mutants are the most sensitive to inhibition

**Figure 1:**
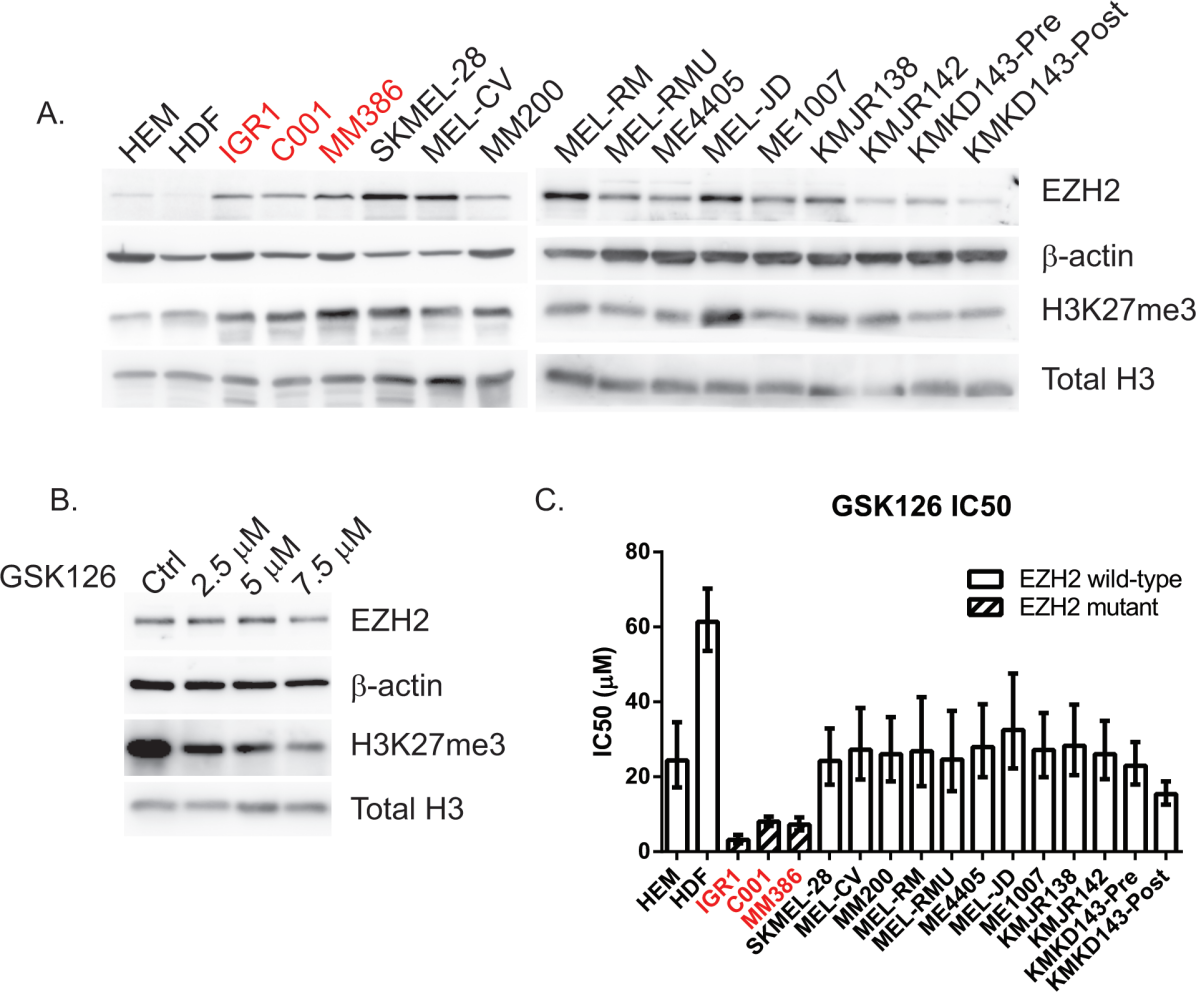
EZH2 and H3K27me3 are overexpressed in both mutant and WT melanoma cell lines but Y646 mutants are more sensitive to EZH2 inhibition Western blot of endogenous EZH2 and the downstream H3K27me3 mark in a panel of melanoma cell lines. EZH2^Y646^ mutants are highlighted in red, human epithelial melanocytes (HEM) and human dermal fibroblasts (HDF) were used as untransformed, normal cells. β-actin was used as a loading control for total cell lysates whereas total histone 3 (H3) was used for purified histones **A.** Dose dependent reduction of H3K27me3 in IGR1^Y646^ mutant cells treated with GSK126 for 48 hr compared to vehicle treated control (DMSO) **B.** IC50 values of GSK126 following 72 hr of treatment using a cell titer glow (CTG) viability assay **C.**

To investigate the role of activated, mutant EZH2 in melanoma biology we collected three melanoma cell lines harboring endogenous EZH2^Y646^ mutations (IGR1, C001 and MM386), a large panel of melanoma cell lines with wild-type (WT) EZH2 and non-transformed melanocytes (HEM) and dermal fibroblasts (HDF). EZH2 mutation status was confirmed by sequencing (Supplementary Figure S1A, S1B) and endogenous levels of EZH2 protein and downstream H3K27 trimethylation were assessed by western blotting (Figure 1A). EZH2 and H3K27me3 were expressed at higher levels in both mutant and WT cells compared to untransformed cells. To test whether GSK126 reduced the methyltransferase activity of EZH2, a time course experiment was performed in the IGR1 mutant cell line. A dose-dependent reduction in H3K27me3 was observed by western blot 48 hr after drug treatment without affecting EZH2 protein levels (Figure 1B). This H3K27me3 reduction was confirmed in 4 other cell lines (Figure 3G) both EZH2 mutant and WT. To establish the sensitivity of cells to the drug, the IC50 of GSK126 was determined for all cell lines illustrated in Figure 1C and Supplementary Figure S1C. The EZH2^Y646^ mutant cell lines were the most sensitive to GSK126 inhibition ranging from 3.2–8.0 μM, whereas normal HDF cells were the least sensitive at 61.4 μM (Figure 1A, Supplementary Figure S1C) indicating selectivity for the mutant enzyme.

### GSK126 reduces proliferation and causes a G2/M cell cycle arrest in EZH2 mutant and wild-type melanoma

**Figure 2:**
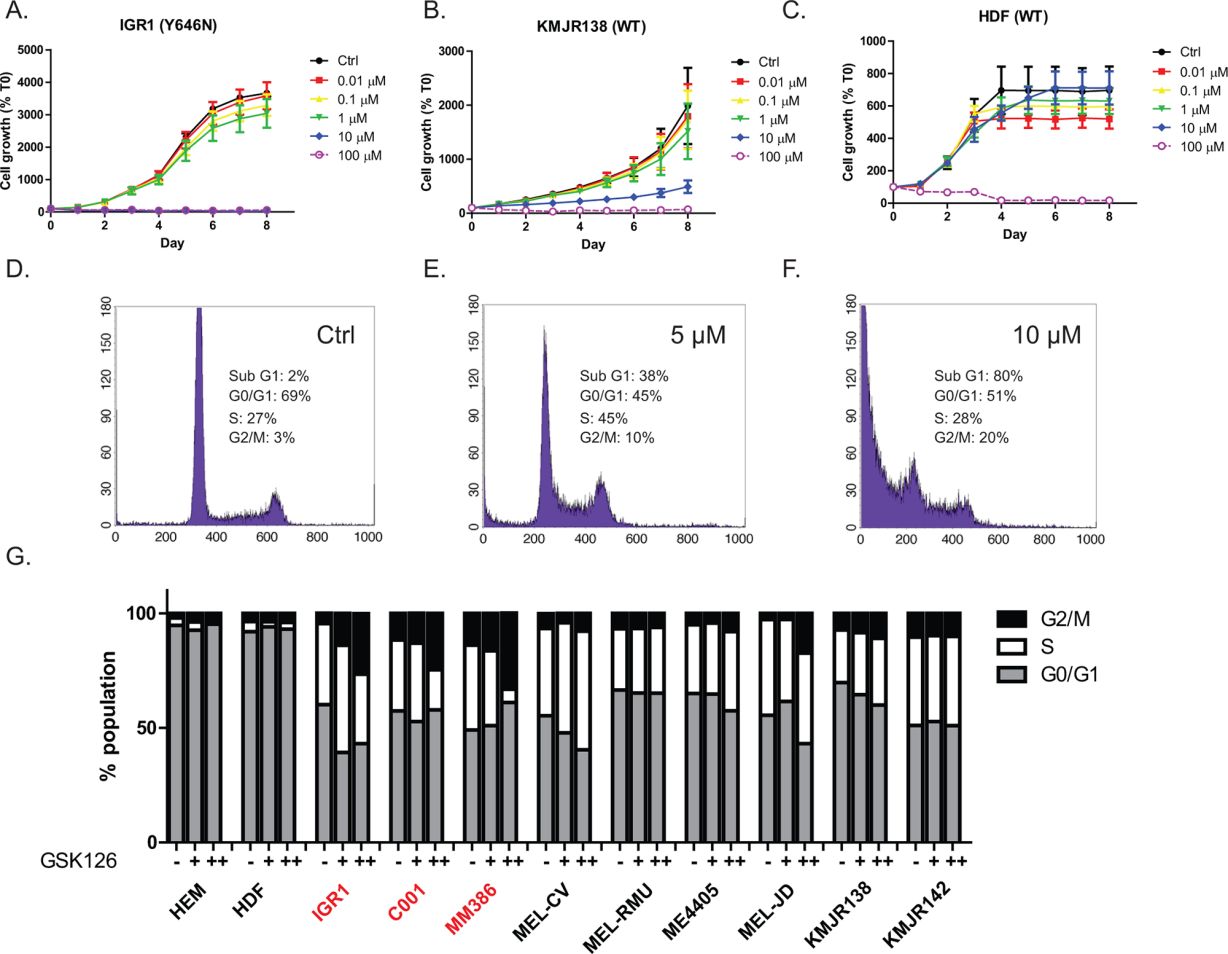
GSK126 inhibits proliferation and causes G2/M cell cycle arrest in EZH2 mutant and WT melanoma Dose dependent effects on proliferation over time in EZH2 mutant **A.** wild-type **B.** and normal cells **C.** using incucyte. Growth is expressed as a percentage of images acquired at time zero (T_0_). Histograms representing DNA content following 72 hr of GSK126 treatment in IGR1 cells **D–F.** Phases of the cell cycle in different cell types are shown in **G.** treated with either 5 μM (+) or 10 μM (++) GSK126. EZH2^Y646^ mutants are highlighted in red.

To observe the influence of GSK126 on cell growth, a proliferation assay was undertaken using IncuCyte. Proliferation of all cell types, regardless of EZH2 mutation status, was inhibited at a drug concentration of 10 μM as early as 3 days after treatment (Figure 2A, 2B, Supplementary Figure S2B), however normal HDF cells remained unperturbed (Figure 2C, Supplementary Figure S2B). Cell cycle analysis of IGR1 (EZH2^Y646N^) cells treated with GSK126 showed a dose dependent accumulation of cells in the G2 phase and a concomitant reduction of cells in the G0/G1 phase, indicative of G2 arrest following 72 hr drug treatment (Figure 2D–2F). A similar pattern was observed in the other EZH2 mutant and some EZH2 WT cell lines but not in normal cells (Figure 2G).

### GSK126 causes AIFM1 release from the mitochondria, inducing caspase independent apoptosis

**Figure 3:**
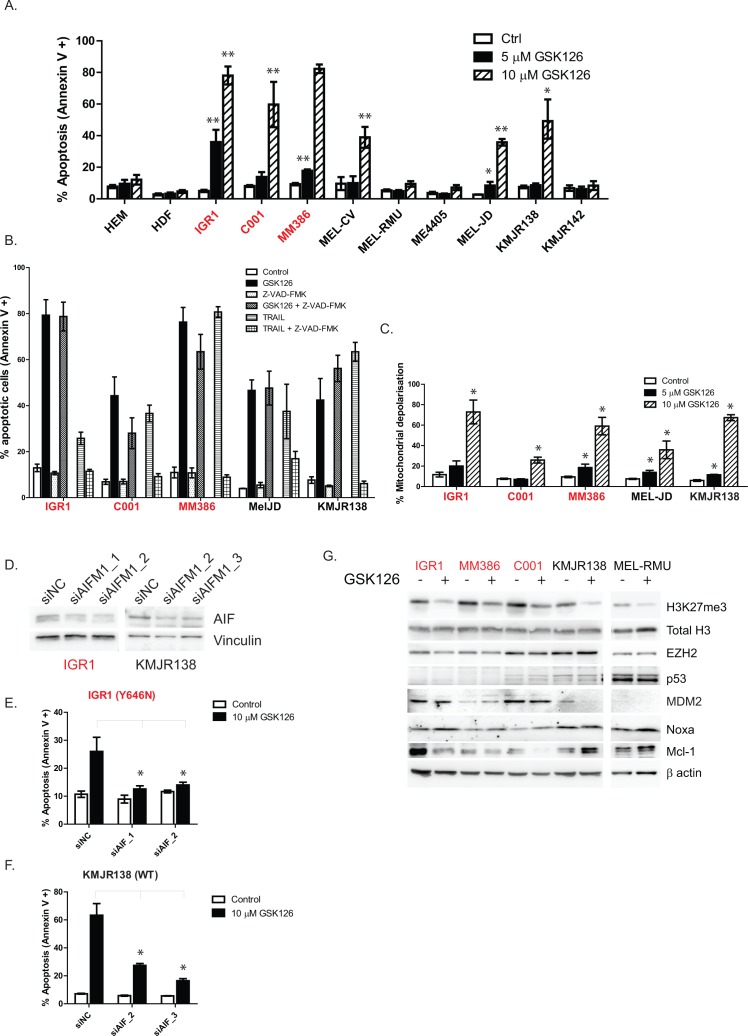
GSK126 induces apoptosis in a caspase independent manner, involves mitochondrial depolarization and AIFM1 release Cells were treated with different doses of GSK126 for 72 hr and apoptosis (combined early and late) was measured by annexin V staining with PI exclusion via flow cytometry **A.** GSK126 mediated apoptosis could not be prevented by the addition of a pan caspase inhibitor (Z-VAD-FMK) that could prevent TRAIL induced cell death, used as a control **B.** Mitochondrial depolarization was observed 48 hr after drug treatment, indicated by JC1 staining and flow cytometry **C.** Knockdown of apoptosis inducing factor (AIFM1) using two different siRNAs **D.** was able to protect cells from GSK126 mediated apoptosis in both EZH2 mutant **E.** and wild-type **F.** cells. Cell lines were screened for apoptotic proteins following 48 hrs of drug treatment using 7.5 μM GSK126 **G.** EZH2^Y646^ mutants are indicated in red.

Cell cycle analysis revealed an accumulation of cells in the sub-G1 phase following drug treatment, indicative of apoptosis (Figures 2E, 2F). To confirm this observation we analyzed cell death using Annexin V/PI staining and flow cytometry, 72 hr after treatment with GSK126 (Figure 3A). Consistent with IC50 values, the highest levels of apoptosis were observed in the EZH2^Y646^ mutant cell lines, but also in three WT cell lines (MEL-CV, MEL-JD and KMJR138) that displayed high levels of H3K27me3 (Figure 1A). Other WT and normal cells were unaffected (Figure 3A). To further characterize the type of apoptosis, a pan caspase inhibitor Z-VAD-FMK was added to the cells in combination with GSK126 (Figure 3B). Z-VAD-FMK failed to prevent GSK126 mediated cell death, indicating that apoptosis was caspase independent. Z-VAD-FMK was however able to prevent TRAIL induced cell death, demonstrating the effectiveness of the caspase inhibitor (Figure 3B).

Caspase independent cell death is typically characterized by disruption of the mitochondrial membrane followed by the release of AIFM1 into the cytoplasm. AIFM1 then translocates to the nucleus where it causes DNA fragmentation and apoptosis. To test whether this pathway is involved in GSK126 mediated cell death, JC1 staining was used to detect mitochondrial depolarization in response to drug treatment. Figure 3C shows a dose-dependent increase in mitochondrial depolarization after 48 hr of treatment. Importantly, knockdown of AIFM1 (Figure 3D) was able to rescue two EZH2 mutant (Figure 3E, Supplementary Figure S2C) and two EZH2 WT (Figure 3F, Supplementary Figure S2C) cell lines from GSK126 induced cell death, indicating AIFM1 release is a critical mediator of apoptosis.

To elucidate the key meditators of mitochondrial damage preceding AIFM1 release, a western blot screen of apoptotic proteins was performed (Figure 3G, Supplementary Figure S2A). EZH2 inhibiton caused an increase in the pro-apoptotic protein Noxa, known to be regulated by the tumor suppressor p53, however no change in p53 protein was observed in drug treated cells and even absent in most of the sensitive cell lines (Figure 3G). Low levels of p53 were associated with higher levels of the p53 ubiquitin ligase MDM2, which targets p53 for proteasomal degradation and is prevalent in melanoma cell lines [23]. A striking decrease in the anti-apoptotic protein Mcl-1 was observed in the EZH2 mutant but not WT cell lines (Figure 3G).

### Knockdown of EZH2 has potent inhibitory effects on survival of EZH2^Y646^ mutant and WT melanoma

**Figure 4:**
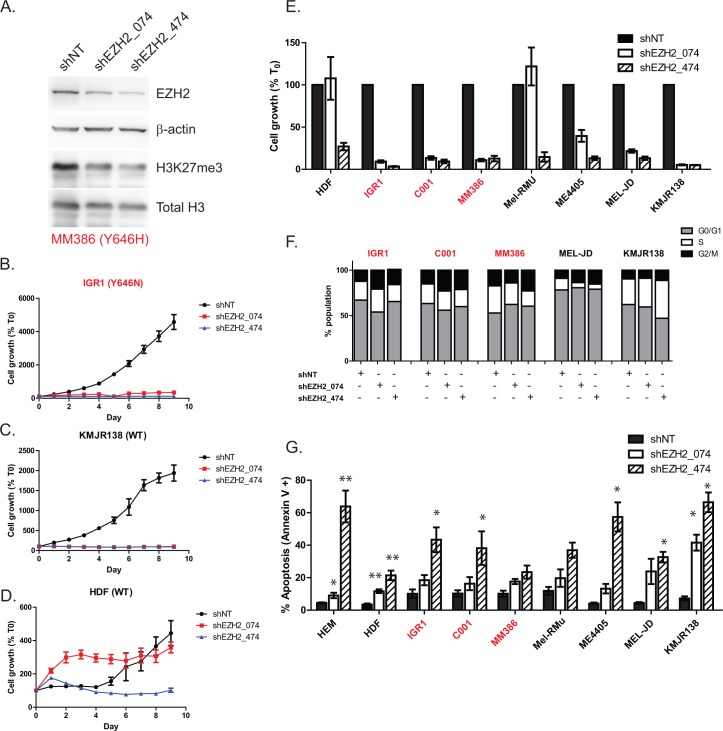
Knockdown of EZH2 phenocopies GSK126 mediated inhibition in melanoma EZH2 protein was depleted using two different lentiviral vectors expressing shRNAs **A.** Following 96 hr of puromycin selection, proliferation was tracked over time using Incucyte in EZH2 mutant **B.** wild-type **C.** or normal cells **D.** Day 8 proliferation values in cell panel with EZH2 knockdown are depicted in **E.** normalized to control values at 100%. Effects on cell cycle are shown in **F.** and apoptosis was measured using annexin V/PI staining **G.** EZH2^Y646^ mutants are highlighted in red.

To confirm GSK126 was mediating its effects via EZH2, stable knockdown of EZH2 was employed using two individual short hairpin (sh) lentiviral vectors (Figure 4A, Supplementary Figure S3A). Although GSK126 does not reduce EZH2 protein, knockdown of EZH2 was shown to deplete levels of H3K27me3 in a similar manner to the drug (Figure 4A). EZH2 knockdown led to a dramatic inhibition of proliferation in all cell types, including WT cells that previously showed resistance to GSK126 (Figure 4B–4E). Consistent with Figure 2G, cells showed a G2/M cell cycle arrest upon EZH2 knockdown, however this was not as pronounced as drug treatment (Figure 4F). Finally, EZH2 knockdown was able to induce apoptosis in all cell types, depending on the degree of depletion (Figure 4G, Supplementary Figure S3A). Consistent with the drug data, apoptosis could not be prevented by the addition of Z-VAD-FMK, indicating that cell death is also caspase independent (Supplementary Figure S3B).

### GSK126 induces cell growth inhibition by de-repression of tumor suppressor gene targets

**Figure 5:**
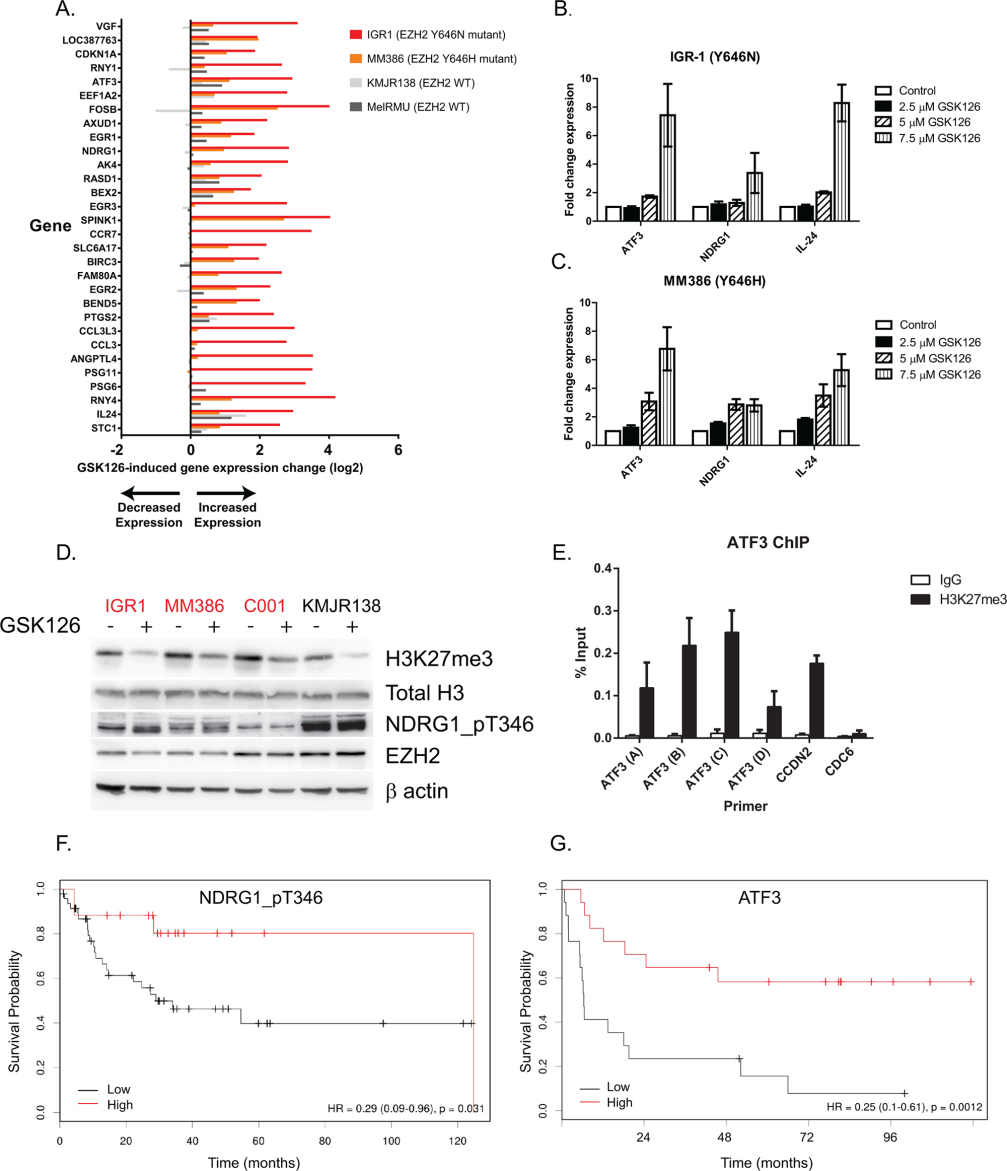
GSK126 causes cell growth inhibition via de-repression of tumor suppressor genes Microarray analysis of the top 30 most differentially expressed genes following 48 hr of 7.5 μM GSK126 treatment. Two sensitive (apoptosis) EZH2^Y646^ mutant cell lines were selected (IGR1 and MM386) along with a sensitive WT (KMJR138) and an insensitive cell line (MEL-RMU) performed in duplicate. **A.** The top candidates with known tumor suppressor gene functions were validated by RT-qPCR in IGR1 **B.** and MM386 **C.** mutant cells. Western blot of cell lines treated for 48 hr with 7.5 μM GSK126 showing the effect on NDRG1_pT346 protein **D.** ChIP-qPCR (*n* = 2) in MM386 cells showing enrichment of H3K27me3 upstream of the transcription start site (primers (A–C) but not downstream (primer D) of the ATF3 gene promotor. CCND2 was used as a positive control and CDC6 as a negative control **E.** Survival analysis (Cox proportional hazard model) of reverse phase protein array (RPPA) data from 31 TCGA melanoma patients divided into high (>0.2963) or low (<0.2963) NDRG1_pT346 protein expression **F.** Survival analysis (Kaplan-Meier) of microarray data from 34 stage III melanoma patients [55] divided into high (upper 75^th^ percentile) or low (lower 25^th^ percentile) ATF3 mRNA expression **G.** Mutants are indicated in red.

To define the mechanism of growth inhibition, RNA expression analysis by microarray was employed to identify potential EZH2 target genes that are de-repressed by GSK126 treatment. Two EZH2^Y646^ mutants (IGR1 and MM386) were treated at two time-points to look for changes in gene expression. The mutants were compared to one partially sensitive (KMJR138) and one relatively insensitive (MEL-RMU) WT cell line to look for genes associated with GSK126 mediated cell death (Figure 5A). After 8 hr of treatment only 13 genes were significantly differentially expressed in the mutants and 2 in the WT cells. This is consistent with the kinetics of H3K27 demethylation that needs to occur before gene expression can be upregulated. After 48 hr of treatment 681 genes were significantly differentially expressed in the mutants, compared to 13 in WT cells (Data not shown). Genes that were common to both EZH2 mutants and WT cells included IL24 (Interleukin 24), GDF15 (Growth Differentiation Factor 15), CCND2 (Cyclin D2) and ULBP2 (UL16 Binding Protein 2). The top 30 most significant gene changes in the mutant cell lines are displayed in Figure 5A and all involved upregulation; as expected given the repressive nature of H3K27me3. Several genes were selected for validation by qRT-PCR, based on their previously well-defined tumor suppressor roles. Figure 5B and 5C confirms a dose-dependent up-regulation of these genes after 48 hr of drug treatment that was more prominent in the EZH2 mutant cell lines compared to WT (Supplementary Figure S4A). This included ATF3 (Activating Transcription Factor 3) and metastasis suppressor NDRG1 (N-Myc Downstream Regulated Gene 1) that also showed an upregulation in protein expression (phosphorylated at threonine 346) with GSK126 in two EZH2 mutant cell lines by western blot (Figure 5D). ChIP studies revealed the promotor region upstream of the ATF3 transcription start site was enriched for H3K27me3, supporting the likelihood of ATF3 being targeted by EZH2 (Figure 5E). Supporting their role as tumor suppressors, low expression of NDRG1_pT346 or ATF3 was associated with a significant decrease in survival of patients in the TCGA melanoma dataset (Figure 5F and 5G). Additionally RPPA data from the TCGA [14] showed that melanoma patients with activated EZH2 (somatic mutation, copy number gains or mRNA activation) show significantly decreased levels of NDRG1_pT346, supporting the hypothesis that aberrant EZH2 activity represses tumor suppressor genes in cancer (Supplementary Figure S4B).

### GSK126 inhibits the growth of 3D spheroid cultures in EZH2^Y646^ mutant and WT melanoma

**Figure 6:**
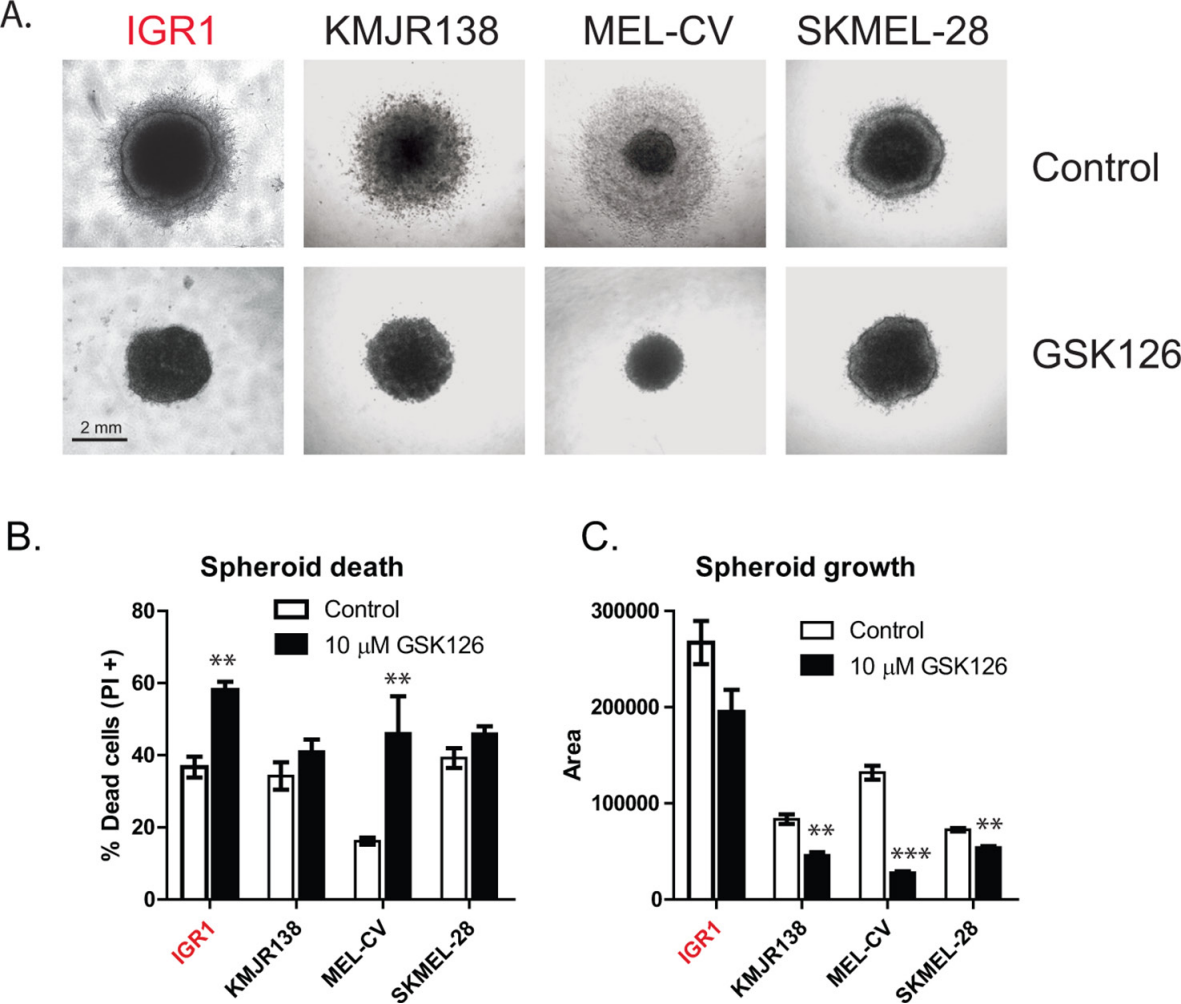
GSK126 inhibits the growth of melanoma spheroids growing in 3D culture The growth of 3D spheroid cultures embedded in collagen was assessed after 72 hr of drug treatment, all images were acquired at 4 × magnification with scale bars representing 2 mm **A.** Spheroids were dissociated with collagenase treatment and cell death measured by PI staining **B.** or the spheroid area was measured using ImageJ software, encompassing the entire spheroid to the outside edge of live cells **C.** Mutants are indicated in red.

Spheroid models were used to test the effectiveness of EZH2 inhibition in 3D melanoma cultures. Cells were grown for 3 days on top of agar to assess anchorage independent spheroid formation. IGR1, KMJR138, MEL-CV and SKMEL-28 spheroids were selected to be embedded in collagen, based on their ability to hold together during this process (data not shown). Following 3 days of growth, a halo of live cells can be seen in the control spheroids that invade the surrounding collagen, a process that is prevented by GSK126 treatment (Figure 6A). This was confirmed by PI staining and flow cytometry that showed a higher percentage of dead cells in spheroids following EZH2 inhibition and reduced overall size (Figure 6B, 5C).

## DISCUSSION

The current report is the first to evaluate the potential of EZH2 inhibitors as a therapeutic strategy in human melanoma. In normal development, EZH2 expression typically declines after birth and remains low in many adult mammalian tissues [24], with the exception of cancer. This also appears to be the case in melanoma; in which EZH2 and H3K27me3 were found to be upregulated in both EZH2^Y646^ mutant and WT cell lines, compared to normal cells. Consistent with previous studies [25], the highest levels of methylation were not necessarily found in cells that expressed the highest level of EZH2 (Figure 1A), suggesting that the methylation activity of EZH2 is perhaps more important than total levels of EZH2 protein. Indeed WT cells (MEL-CV, MEL-JD and KMJR138) with high H3K27me3 displayed increased apoptosis and spheroid growth inhibition (Figure 3A, 6). Further studies are needed to assess whether H3K27me3 may represent a better biomarker than EZH2 in predicting response to EZH2 inhibitor therapy and survival outcomes.

Treatment with the EZH2 inhibitor GSK126 effectively reduced the levels of H3K27me3 in melanoma cell lines in a dose dependent manner, leading to a reduction in proliferation, cell cycle arrest and apoptosis. Not only was this evident in 2D cultures but also in 3D spheroid models growing in collagen that more accurately recapitulates the tumor microenvironment [26]. The 3D cultures showed that GSK126 treatment reduced infiltration of cells into collagen which is consistent with the increased mobility reported in A375 melanoma cells transfected with EZH2 [27]. Consistent with lymphoma studies, the highest levels of growth inhibition was observed in melanoma cell lines harboring an activating EZH2^Y646^ mutation.

Induction of apoptosis by GSK126 was caspase independent and involved the release of AIFM1 from mitochondria, as shown by inhibition of apoptosis upon AIFM1 knockdown. The signals leading to AIFM1 release in the GSK126 treated cells remain unclear but were associated with an increase in the pro-apoptotic protein Noxa. Noxa is known to bind to the anti-apoptotic protein Mcl-1 thus promoting its degradation and release of activated Bak to trigger mitochondrial apoptosis [28]. The increase in NOXA and decrease in Mcl-1 suggests combining EZH2 inhibition with a BH-3 mimetic such as ABT-737 could increase cytotoxic effect. ABT-737 inhibits Bcl-2; Bcl-w and Bcl-XL but melanoma cells are typically protected from the cytotoxic effects of ABT-737 by high levels of Mcl-1. The combination of Noxa overexpression or Mcl-1 decrease strongly sensitized melanoma cells to ABT-737 [29].

Stable knockdown of EZH2 via lentiviral mediated shRNA produced remarkably similar changes to that of GSK126, highlighting the specificity of the drug. This included striking inhibition of proliferation, cell cycle arrest and apoptosis that correlated with the level of EZH2 knockdown by different shRNA. Apoptosis was similar to that induced by GSK126 in being caspase independent. The effect of knockdown was not restricted to EZH2 mutants but also WT cells that showed insensitivity to GSK126. This is likely because knockdown of EZH2 protein may disrupt interactions between EZH2 and other members of the PRC2 complex, instead of merely blocking methyltransferase activity by competitive inhibition. This implies that the latter strategy is perhaps more appropriate for targeted therapy and may explain why earlier inhibitors that degrade EZH2 protein; such as DZNep have not progressed to clinical trials, perhaps due to lack of specificity for H3K27me3 [30].

Although the basis for increased sensitivity of EZH2 mutants is understood, it was not clear why some melanomas with WT EZH2 were also partially sensitive. It was reported that BRAF^V600^ mutated melanoma had aberrant DNA methylation activity and that BRAF inhibition could reverse the DNA methylation of some genes [16]. Additionally EZH2 and BRAF mutations show a tendency toward co-occurrence [13]. In view of this, we examined whether there may have been an association with BRAF status and EZH2 or H3K27me3 levels but were unable to show a significant association. Nevertheless melanoma cultures (KMKD) obtained from a patient before and after treatment with the BRAF inhibitor, vemurafenib showed a decrease in levels of EZH2 protein in the treated cells and a decrease in the GSK126 IC50 values. Further studies are needed to define the relationship between EZH2 and MAP kinase signaling but these results suggest that combining BRAF and EZH2 inhibitors may provide an effective treatment strategy for melanoma.

Given the role of EZH2 in down regulation of suppressor genes we focused on genes showing increased expression in microarrays after treatment with GSK126. We identified CDKN1A that encodes the cyclin dependent kinase inhibitor p21, which activates multiple tumor suppressor pathways [31]. CDKN1A has been previously implicated as an EZH2 target gene in melanoma and other cancers and can promote G2/M arrest [32, 33]. Scrutiny of the top 30 genes identified a number of novel EZH2 target genes with possible tumor suppressor gene roles, including the ATF3 transcription factor; validated by ChIP-qPCR. Despite pleiotropic effects, this stress induced gene is upregulated by many anti-tumorigenic compounds, and over-expression results in apoptosis and reduced metastatic potential, in a cell-type dependent manner [34]. It is known to negatively regulate cyclin D1 [35] and loss of ATF3 was recently shown to promote prostate cancer in a transgenic mouse model [36]. Importantly, the ATF4-ATF3-CHOP apoptotic cascade has been associated with release of AIFM1 from mitochondria [37, 38], activation of Noxa and repression of Mcl-1 [39], a prominent feature in our study.

Another potential EZH2 target gene with growth inhibitory roles was the metastasis suppressor NDRG1. Phosphorylation of NDRG1 at Thr346 has been shown to repress NF-κB signaling and CXC cytokines, critical to the regulation of metastatic processes such as proliferation, adhesion and chemotherapy resistance [40]. The NDRG1 promoter is hypermethylated and repressed in many types of cancers and overexpression studies in multiple cell lines have demonstrated growth inhibitory effects both *in vitro* and *in vivo* [41]. Additionally, NDGR1 has been implicated as an important inhibitor of TGF-β induced epithelial to mesenchymal transition (EMT); a critical precursor of metastasis [42]. Studies have demonstrated a key role for EZH2 in driving EMT [15, 43] a process that may be thwarted by blocking EZH2 and in turn upregulating the NDRG1 suppressor. The importance of ATF3 and NDGR1 as tumor suppressors in melanoma was supported by the analysis of melanoma patient survival data in the TCGA that low levels of these genes were associated with poor survival.

Several of the genes upregulated by GSK126 suggest that it may also have effects on immune responses. The upregulation of genes implicated in anti- tumor immune responses was observed, including CCL3 (Chemokine Ligand 3), the Natural Killer cell ligand; ULBP2 and IL24. IL24 was originally implicated in terminal differentiation of human melanoma cells and subsequently shown to selectively kill cancer cells, inhibit tumor growth and invasion and metastasis *in vitro* and *in vivo* [44]. Importantly, IL24 exposure induced apoptosis by reduction of pro-apoptotic Bcl2 proteins [45] and caused a G2/M cell cycle arrest [46]; a similar effect observed in melanoma cell lines treated with GSK126. IL24 was one of the few significantly upregulated genes in both EZH2 WT and EZH2 mutant cells (Figure 5A–5C), thus broadening the range of potential therapeutic benefit of GSK126. Further studies are needed to validate these genes as bonafide EZH2 targets whose expression is repressed by aberrant methylation in melanoma.

Collectively these studies are the first to demonstrate that human melanoma cells with activating mutations in EZH2 display a critical dependency on this enzyme for their growth and survival. They provide further support for EZH2 as a promising therapeutic target in melanoma treatment, especially in EZH2^Y646^ mutants. Further studies are needed to define the basis for sensitivity of EZH2 WT melanoma to EZH2 inhibition and whether gene signatures can be used to predict melanomas that are sensitive to EZH2 inhibitors.

## MATERIALS AND METHODS

### Cell lines

EZH2^Y646^ mutant melanoma cell lines C001 and MM386 were from Dr. Chris Schmidt, QIMR, Brisbane, Australia. IGR1 cells were from Dr. David Adams, WTSI, Cambridge, UK. The EZH2^Y646^ mutation was confirmed by Sequenom genotyping or Sanger sequencing. Human melanoma cell lines Mel-RMU, SKMEL-28, MEL-RM, MEL-JD, ME1007, MM200 and ME4405 have been described previously [47, 48]. Untransformed, human dermal fibroblasts (HDF) were purchased from the American Type Culture Collection (ATCC, Manassas, VA, USA) and human epithelial melanocytes (HEM) were purchased from Life Technologies. Cells were cultured in Dulbecco's modified Eagle medium (DMEM) supplemented with 10% fetal calf serum (FCS; AusGeneX, Brisbane, Qld, Australia) and Pen/Strep (Sigma, St Louis, MO, USA). HEM were cultured in M254 containing HGMS and all cells were maintained at 37°C in 5% CO_2_. In addition, primary melanoma cultures were obtained from a patient enrolled in a BRAF inhibitor (vemurafenib) study, both prior to (KMKD142-Pre) and during relapse (KMKD142-Post) from treatment with the drug (Lai, 2012, Nguyen 2001). All cells lines were authenticated by STR genotyping.

### Immunoblotting

Cell pellets were lysed with radioimmunoprecipitation (RIPA) buffer and western blotting was conducted as described previously (Irvine, 2010). For detection of histone methylation, acid extraction was used to purify histones as described previously [49]. Total protein was determined using a BCA assay (Bio-Rad, Hercules, CA, USA). Labeled bands were detected by Clarity ECL kit (Bio-Rad) and images were captured by the Fujifilm LAS-4000 image system.

### Antibodies

Antibodies used were as follows: EZH2 (Cell Signaling, #5246, Danvers, MA, USA), H3K27me3 (Active motif, #61017, Carlsbad, CA, USA), Beta Actin (Sigma, #AC-74), Histone 3 (AbCam, Cambridge, UK, ab1791), AIF (Santa Cruz, #sc-5586, Dallas, Texas USA), NDRG1_pT346 (Cell Signaling #3217), p53 (Santa Cruz, #sc-126), Noxa (Imgenex, Littleton, CO, USA, #114C307.1), Mcl-1 (BD Biosciences, #559027). Additional antibodies are listed in Supplementary Table S2.

### Chemical reagents and gene silencing

GSK126 was purchased from Medchemexpress (New Jersey, USA) and dissolved in DMSO that was used as the vehicle control in all experiments. For knockdown studies, siRNA molecules were purchased from Shanghai Gene Pharma (Shanghai, China): Negative control (5′-UUCUCCGAACGUGUCACGUTT-3′), AIFM1_1 (5′-GGAACAUCUUUAACCGAAUTT-3′), AIFM1_2 (5′-GCAGUGGCAAGUUACUUAUTT), AIFM1_3 (5′-CGUACUGGCAUCAGUCAAUTT-3′). Cells were reverse transfected using Lipofectamine RNAimax according to the manufacturer's instructions 48–72 hr prior to treatment with GSK126. The pan caspase inhibitor Z-VAD-FMK (SM Biochemicals LLC, Anaheim, CA, USA) was used in apoptosis assays.

### Lentiviral production and transfection

Lentiviral vectors (pLKO.1) were purchased from Openbiosystems and calcium phosphate transfection of HEK293T packaging cells was used to make virus as described previously [50]. A non-targeting (shNT; #RHS6848) vector was used as a control. The target sequence of the vectors is as follows: shEZH2_074 (5′-GCTAGGTTAATTGGGACCAAA-3′), shEZH2_ 474 (5′-CAACACAAGTCATCCCATTAA-3′). Concentrated viral titers were determined using the QuickTiter Lentivirus Quantitation Kit (Cell Biolabs Inc, San Diego, CA, USA). Cells were transfected with virus in the presence of Polybrene (8 μg/mL) and selected with puromycin (1 μg/mL) 48 hr after transfection for an additional 96 hr.

### Cell viability and proliferation assays

Cells were seeded in white 384 well plates in at a pre-determined optimal seeding density. After 24 hr cells were treated with a 10-fold serial dilution of GSK126 or 0.1% DMSO (vehicle control). Following 3 days of incubation cells were lysed and viability was measured using CellTiter-Glo (CTG, Promega) luminescence detection using a Perkin Elmer Wallac 1420 VICTOR^2^ plate reader. CTG values were normalized to a percentage of control cells and plotted as a dose-response curve. The concentration of GSK126 required to inhibit 50% of cell growth (IC50) was determined. For proliferation assays an IncuCyte 2011A device captured images of cells growing in a 96-well plate at regular intervals and mean confluence was calculated. Untreated cells were imaged to determine the starting number of cells at time zero (T_0_) and data expressed relative to T_0_ in response to drug treatment over time.

### Cell cycle analysis and apoptosis assays

Distribution of cell cycle phases were determined by PI nuclear staining and flow cytometry. Cells were seeded and treated after 24 hr with Control (0.1% DMSO), 5 μM or 10 μM GSK126. After 72 hr cells were harvested, washed and stained with PI staining solution. Samples were evaluated using a FACSCalibur flow cytometer (BD Biosciences) and data were analyzed using Modfit software. Cell death was determined using Annexin V/PI staining according to the manufactures instructions (BD) and analyzed by flow cytometry with CellQuestPro software.

### Gene expression profiling

Cells were seeded in 75 cm^2^ flasks in duplicate and treated the following day with DMSO or 7.5 μM GSK126. Following 8 and 48 hr incubation, cells were harvested, washed and pellets were lysed in TRIzol reagent (Invitrogen). Following purification, RNA quality was verified using the Agilent 2100 Bioanalyser (Agilent Technologies, Palo Alto, CA). Raw microarray data was read into R using limma [51]. Quality control was performed by examining the distribution of non-genomic probes and human housekeeping genes. The arrays were normalized using the normal-exponential deconvolution method [52]. Probes which had a detection *p*-value of less than 0.01 on less than two microarrays were removed from the analysis. All linear models and contrasts were fitted with limma and analysis performed on all cell lines comparing DMSO treated cells with GSK126 treatment after 8 or 48 hr.

### Chromatin immunoprecipitation

Chromatin immunoprecipitation (ChIP) was adapted from previously published protocols [53]. Briefly, MM386 cells were fixed with 1% formaldehyde for 10 minutes, glycine added to neutralise the formaldehyde and nuclei prepared from the cells. Immunoprecipitation was performed overnight with antibodies against H3K27me3 (Activ Motif clone MABI0323) or IgG control (DAKO) coupled to magna-ChIP beads (Millipore). Immunoprecipitates were washed 5 times in LiCl buffer and reverse cross-linked overnight at 65°C before being column purified (Qiagen). Real-time PCR was performed using primers (Supplementary Table S1) on an ABI HT7900 Fast Real-Time PCR System (Applied Biosystems, Calsbad, CA) and values were normalised to the amount of input chromatin.

### qRT-PCR

Cells were seeded in 6-well plates and treated the following day with DMSO, 2.5 μM, 5 μM or 7.5 μM GSK126. After 48 hr RNA was extracted from cells using the RNeasyPlus mini prep kit (Qiagen), quantified using a Nanodrop (Thermo Scientific, Wilmington, DE) and 1 μg was reverse transcribed using SuperScriptIII (Invitrogen). cDNA was amplified using the ABI 7900 Real-Time PCR system using Universal PCR Mastermix and Taqman probes (Applied Biosystems) specific for NDRG1 (Hs00608387_m1), ATF3 (Hs00231069_ml), IL-24 (Hs01114274_m1) and normalized to levels of 18s (Hs99999901_s1).

### 3D Spheroid cultures

Cells were grown on top of a layer of 1.5% agarose in 96-well plates to induce anchorage independent spheroid formation. After 72 hr, multiple spheroids were harvested and embedded in 0.5 mL collagen solution (Cultrex bovine collagen 1; Trevigen Inc, Gaithersburg, MD, USA) in a 24-well plate and left to solidify at 37°C. After 15 min 1 mL of media was added on top of the collagen layer, containing 1.5 × concentration of GSK126 or DMSO [54]. Following 3 days of treatment, spheroids were photographed, dissociated via digestion with 1 mg/mL collagenase (Sigma) and stained with PI to measure cell death by flow cytometry. ImageJ software was used to determine the area [29].

### Statistical analysis

Statistical significance was assessed using non-parametric analysis (Mann-Whitney *U*-test) using GraphPad Prism 5.01 unless otherwise indicated in the figure legend. All error bars represent the standard error from 3 independent experiments (*n* = 3), unless otherwise stated. *p* ≤ 0.05 was considered statistically significant.

## SUPPLEMENTARY FIGURES AND TABLES


