# Supplementary material for: Targeting activating mutations of EZH2 leads to potent cell growth inhibition in human melanoma by derepression of tumor suppressor genes
Source: Oncotarget. 2015 Aug 12;6(29):27023–36. doi: 10.18632/oncotarget.4809 (PMC4694971; doi:10.18632/oncotarget.4809)
Supplement: Supplementary file 1 [file oncotarget-06-27023-s001.pdf]

SUPPLEMENTARY FIGURES AND TABLES

A.

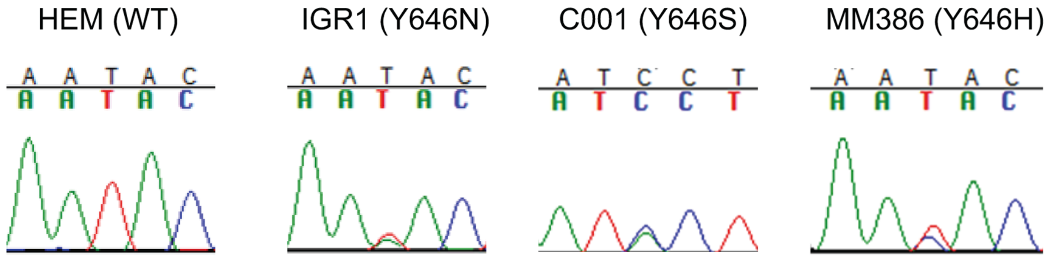

B.

Cell Line	Nucleotide change	Protein change	BRAF	CDKN2A	p14ARF	CDK4	NRAS	PTEN	p53
IGR1	TAC>AAC	Y646N	V600E/M	-	-	-	-	-	-
C001	TAC> TCC	Y646S	WT	WT	WT	WT	Q61K	WT	E258_S261del (EDSS), 771_782delGGAAGACTCCAG
MM386	TAC> CAC	Y646H	V600E	WT(methylated)	WT	WT	WT	HD	WT

C.

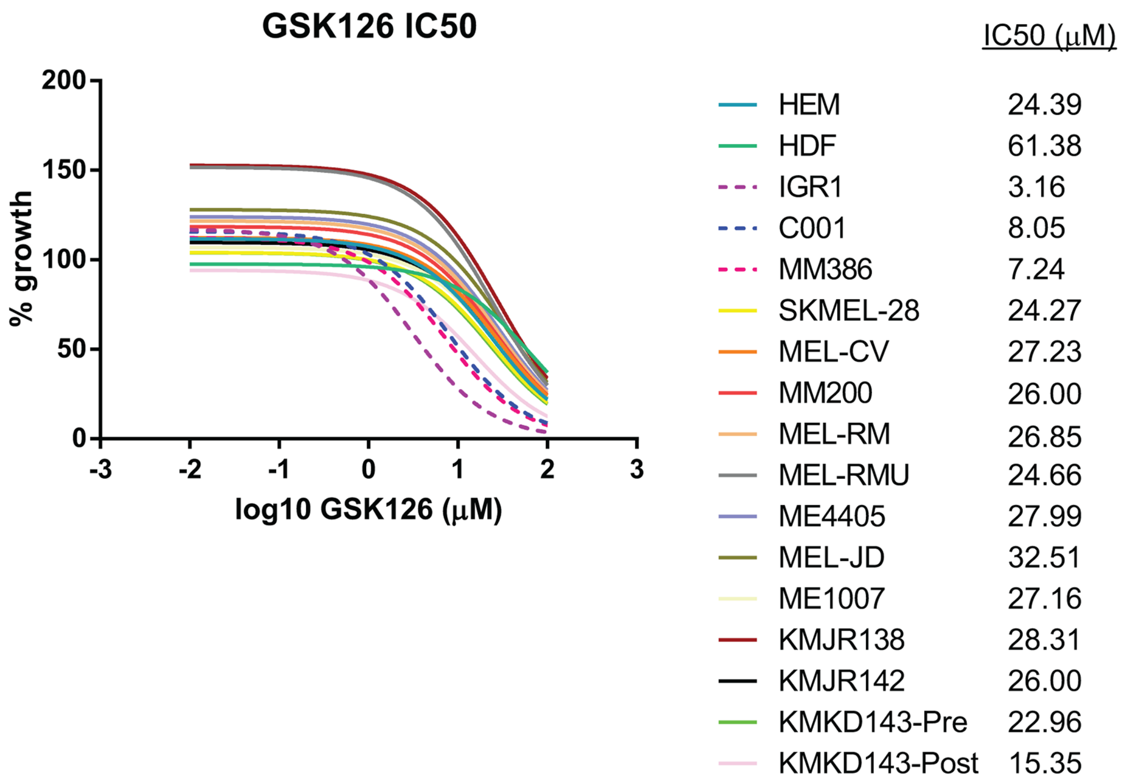

Supplementary Figure S1: Mutation status and IC50 values of cell lines. Sequencing traces of EZH2^{Y646} mutant cell lines identified by Sanger sequencing. **A.** Mutational status of EZH2^{Y646} cell lines **B.** IC50 values of GSK126 against the cell panel **C.**

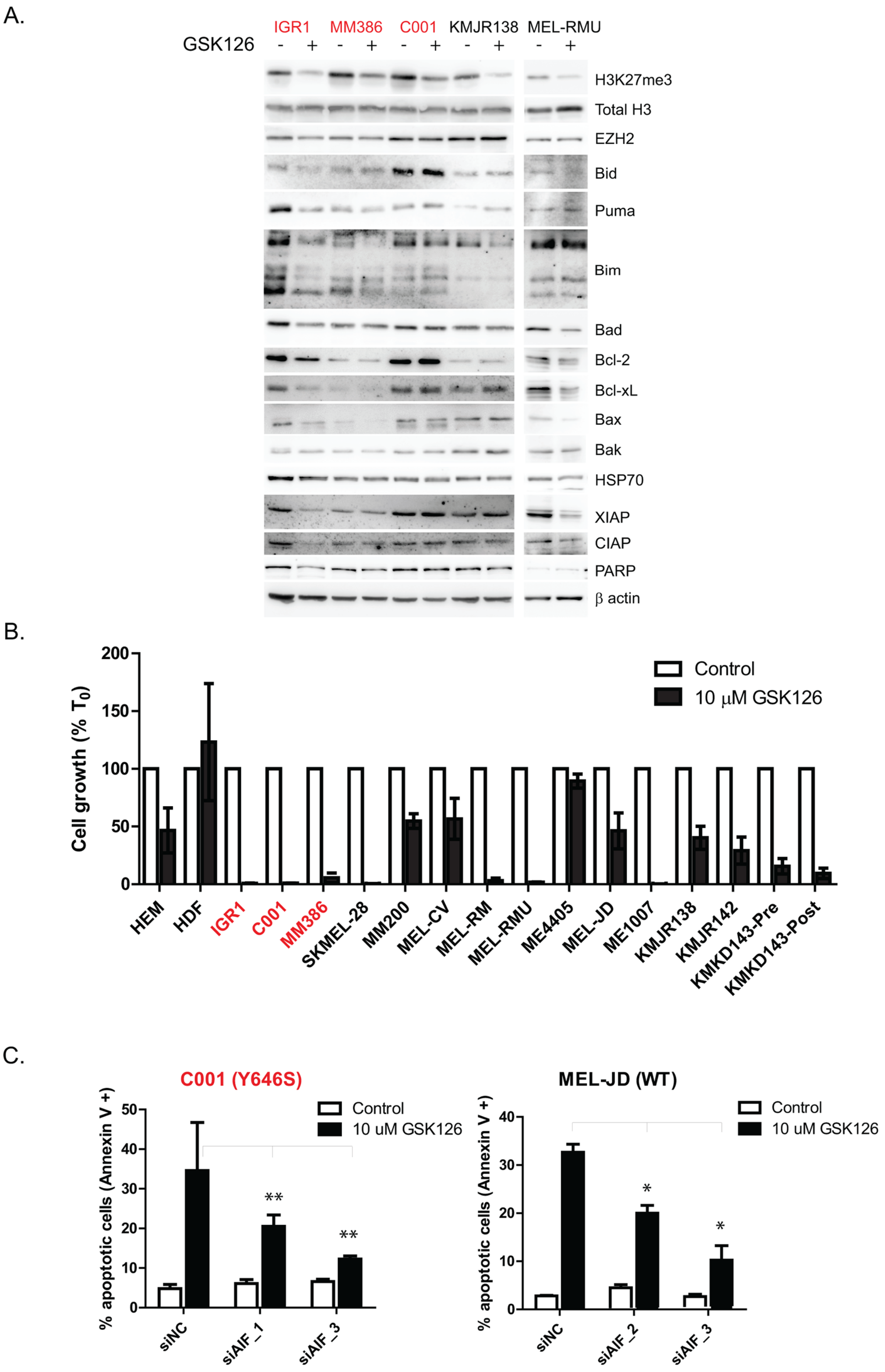

Supplementary Figure S2: Apoptosis screen, Day 8 proliferation values and AIFM1 knockdown in additional cell lines. Western blot of mutant EZH2^{Y646} cell lines (highlighted in red) and WT cells treated with 7.5 μM GSK126 for 48 hr. **A.** Day 8 proliferation values in cell panel treated with GSK126 normalized to control values at 100% **B.** siRNA knockdown of AIFM1 in EZH2^{Y646} mutant (C001) or EZH2 WT (MEL-JD) was able to prevent GSK126 mediated apoptosis **C.** Mutants are indicated in red.

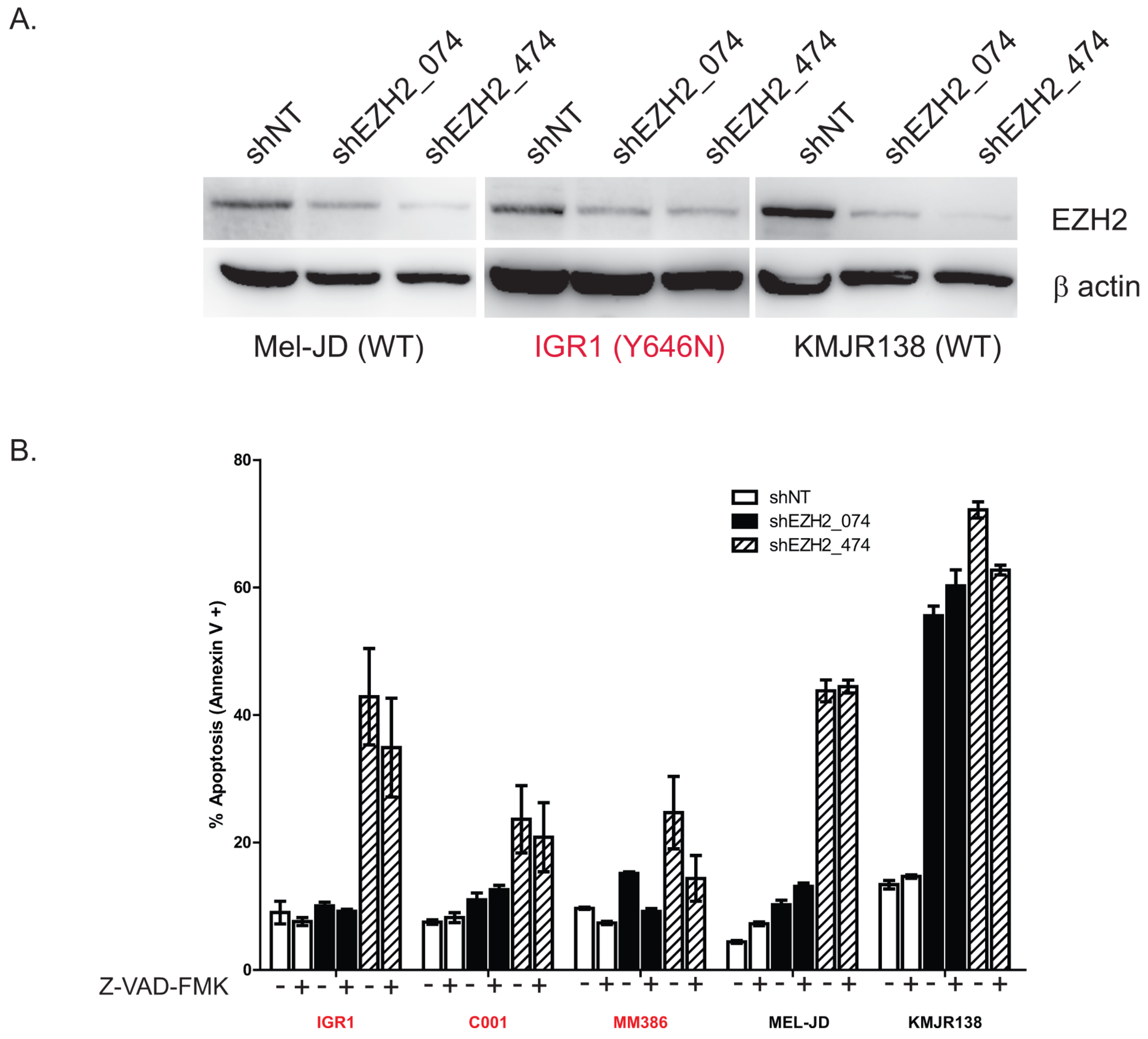

Supplementary Figure S3: EZH2 knockdown and caspase inhibition. Western blot showing knockdown of EZH2 in multiple cell lines using two individual shRNA lentiviral vectors 6 days after transfection including 4 days of puromycin selection. **A.** Pan caspase inhibition in EZH2 knockdown cells 24 hr after transduction was unable to prevent cell death **B.** as in drug treated cells (n = 2). Cells in (B) were not preselected with puromycin as Z-VAD-FMK is known to prevent puromycin cell death. Mutants are indicated in red.

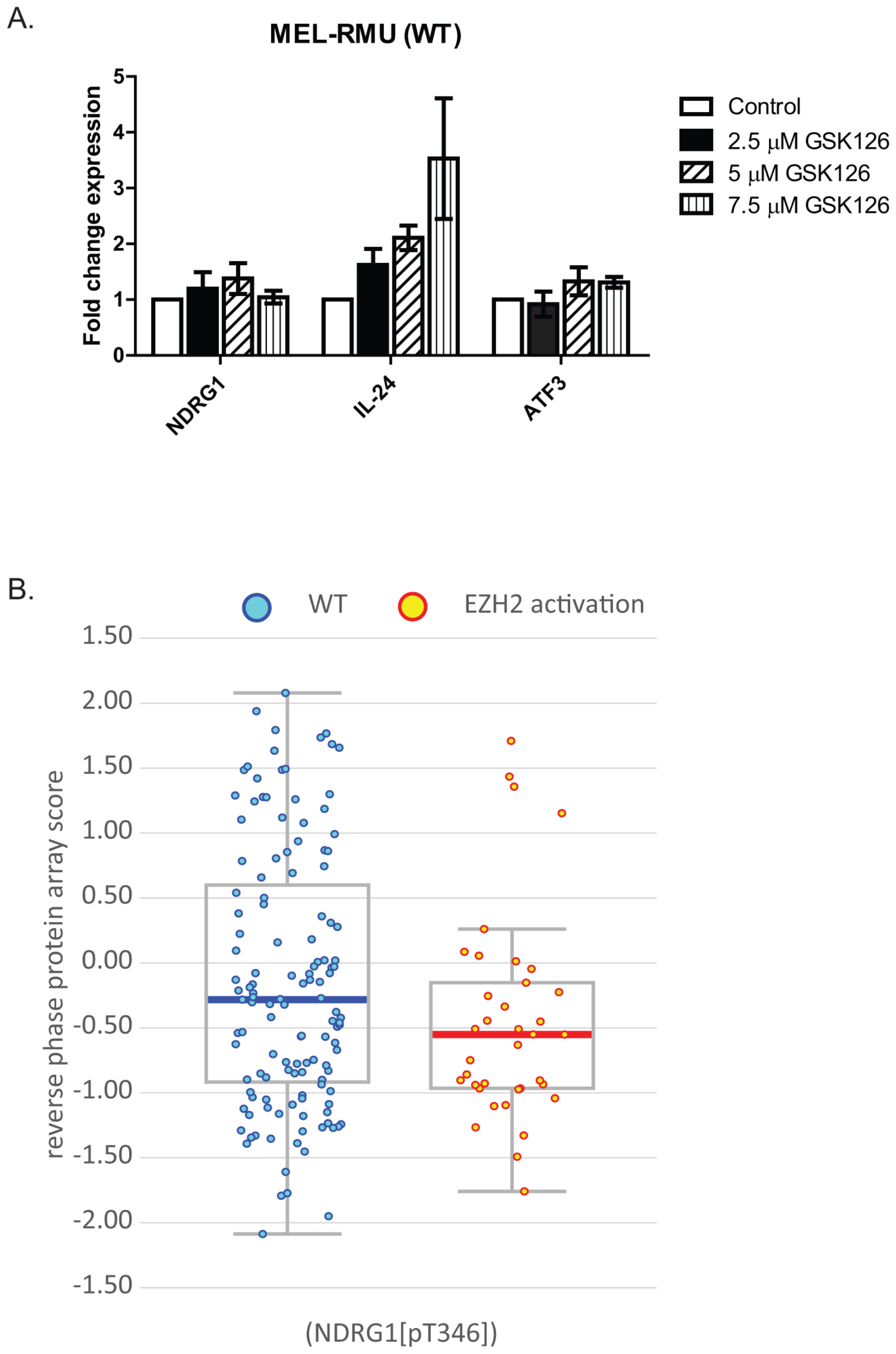

Supplementary Figure S4: Target validation in WT cells and NDRG1_pT346 RPPA data. RT-qPCR of microarray targets in EZH2 wildtype MEL-RMU. **A.** cells treated for 48 hr with different doses of GSK126. Reverse phase protein array (RPPA) data **B.** for NDRG1_pT346 in melanoma patients from TCGA with WT or EZH2 activation (somatic mutations, amplification or mRNA upregulation).

Supplementary Table S1: RT-PCR Primers used in this study

Gene of Interest	Forward Primer	Reverse Primer	Location relative to transcription start site
ATF3 (A)	CTACAGTCACCTTGCGGTGC	GAGGCTGGGAAGGGTAATGG	–10152 to –10041
ATF3 (B)	CCCTCCACTTCATGGCACTG	GTCTTTGCCACAGGAGAGTGATC	–2670 to –2539
ATF3 (C)	GACACCTCTAGTTTCTACCCCAAATT	ACGGTGAAGGGCAGAAACAC	–1453 to –1354
ATF3 (D)	CTTCCTCTTTCAGGACTGTTTG	GGATGATGTTTCAGGCTACTCTG	6018 to 6115
CCND2	TAGGATCCGTTTTGAAGAAGCC	CATTCTGTAGGTGTAGCACGCC	–337 to –273
CDC6	GATTCCCTCCCCCGTTCA	CAATGAGAGAGCCCCAAGTCTT	60 to 124

NB ATF3 transcript NCBI refseq NM_001674

Supplementary Table S2: Antibodies used in this study

Antibody	Catalogue number	Manufacturer
EZH2	5246	Cell Signaling Technology
B-actin	AC-74	Sigma
H3K27me3	61017	Active Motif
Total Histone 3	ab1791	AbCam
AIF	sc-5586	Santa Cruz
NDRG1_pT346	3217	Cell Signaling Technology
p53	sc-126	Santa Cruz
Bim	C34C5	Cell Signaling Technology
Noxa	114C307.1	Imgenex
Mcl-1	559027	BD Bioscience
Bid	2002S	Cell Signaling Technology
Puma	4976	Cell Signaling Technology
Bad	610392	BD Bioscience
Bcl-2	C-2, sc-7382	Santa Cruz
Bcl-xL	H-5, sc-8392	Santa Cruz
Bax	2772	Cell Signaling Technology
Bak	G-23, sc-832	BD Bioscience
HSP70	4872	Cell Signaling Technology
XIAP	610716	BD Bioscience
CIAP	AF 8181	R&D
PARP	F-2, sc-8007	Santa Cruz
MDM2	H-221	Santa Cruz
